# Regulatory and Interacting Partners of PDLIM7 in Thyroid Cancer

**DOI:** 10.3390/curroncol30120761

**Published:** 2023-12-13

**Authors:** Kristiana Rood, Celina Romi Yamauchi, Umang Sharma, Ria T. Laxa, Collin Robins, Gerardo Lanza, Kidianys Sánchez-Ruiz, Aminah Khan, Hae Soo Kim, Andrea Shields, Kari Kennedy, Saied Mirshahidi, Mia C. Perez, Anthony Firek, Iqbal Munir, Alfred A. Simental, Salma Khan

**Affiliations:** 1 Division of Biochemistry, Loma Linda University School of Medicine, Loma Linda, CA 92350, USA; krood@students.llu.edu (K.R.); romi.yamauchi@gmail.com (C.R.Y.); rtlaxa@dons.usfca.edu (R.T.L.); cdrobins@students.llu.edu (C.R.); gerardogomezlanza@gmail.com (G.L.); 121ksanchez@uccaribe.edu (K.S.-R.); kaminah772@gmail.com (A.K.); haesookim@students.llu.edu (H.S.K.); 2 Division of Otolaryngology, Loma Linda University Health, Loma Linda, CA 92354, USA; kdkennedy@llu.edu (K.K.); miperez@llu.edu (M.C.P.); asimenta@llu.edu (A.A.S.); 3 Center for Health Disparities & Molecular Medicine, Loma Linda University School of Medicine, Loma Linda, CA 92350, USA; 4 School of Public Health, Loma Linda University, Loma Linda, CA 92354, USA; usharma@students.llu.edu; 5 Department of Pathology & Human Anatomy, Loma Linda University School of Medicine, Loma Linda, CA 92350, USA; ashields@llu.edu; 6 Loma Linda University Cancer Center, Loma Linda, CA 92354, USA; smirshahidi@llu.edu; 7 Comparative Effectiveness and Clinical Outcomes Research Center (CECORC), Riverside University Health System, 26520 Cactus Ave, Moreno Valley, CA 92555, USA; a.firek@ruhealth.org; 8 Department of Endocrinology, Riverside University Health System, 26520 Cactus Ave, Moreno Valley, CA 92555, USA; iqbalmunir2@gmail.com; 9 Department of Internal Medicine, Loma Linda University School of Medicine, 11085 Campus St, Loma Linda, CA 92350, USA

**Keywords:** thyroid cancer, Enigma, let-7g, PI3K/AKT, MDM2, BMP-1

## Abstract

Enigma protein, encoded by the PDLIM7 gene, is overexpressed in thyroid cancer in a stage-dependent manner, suggesting a potential involvement in the initiation and progression of thyroid cancer. The Enigma interacts with several cellular pathways, including PI3K/AKT, MDM2, and BMP-1. The Enigma is regulated by microRNAs. Specifically, we showed that the Enigma protein upregulation corresponds to the downregulation of Let-7 family genes. There is limited research on the interactions and regulation of the Enigma with other proteins/genes in thyroid cancer tissues, indicating a gap in current knowledge. Our aim is to establish the Enigma as a biomarker. We also aim to study the interacting partners of the Enigma signaling pathways and their probable miRNA regulation in thyroid cancer progression. Using Western blotting, densitometric analysis, immunoprecipitation (IP), and reverse IP, we detected the protein expression and protein–protein interactions in the corresponding papillary thyroid carcinomas (PTCs). Utilizing real-time qPCR assay and Pearson’s correlation test, we highlighted the correlation between PDLIM7 and Let-7g gene expression in the same tissues. The results showed the differential upregulations of the Enigma protein in different stages of PTCs compared to benign tissues along with AKT, VDR, BMP-1, and MDM2 proteins. Loss of DBP was observed in a subset of PTCs. Strong interactions of the Enigma with PI3K/AKT and MDM2 were noted, along with a weaker BMP-1 interaction. Pearson’s correlation coefficient analysis between PDLIM7 and let-7g gene expression was significant (*p* < 0.05); however, there was a weak inverse correlation (r = −0.27). The study suggests the potential utility of the PDLIM7-qPCR assay as a biomarker for thyroid cancer. The Enigma’s interactions with key signaling pathways may provide valuable insights into the development of thyroid cancer. The study contributes to understanding the molecular mechanisms involving the Enigma protein in thyroid cancer and highlights its potential as a biomarker.

## 1. Introduction

Thyroid cancer is known to demonstrate calcification which, in turn, suggests that the processes that promote calcification in nonmalignant tissue may have a role in oncogenesis in thyroid cancer. A key osteogenic protein in normal bone formation, the Enigma, may have a role as either an oncogenic or tumor suppressor gene depending on the cancer type. The Enigma has been colocalized with bone morphogenetic protein 1 (BMP-1), which is known to promote calcification and carcinogenesis in thyroid cancer [1]. Our previous study demonstrated that the Enigma is strongly expressed in thyroid cancer tissues, and a higher expression is associated with tumor size and lymph node involvement [1]. The Enigma is overexpressed in cancer tissues in a stage-dependent manner but is not expressed in benign or normal tissues [1]. The results of our studies suggest that the Enigma may play a key role in the development of thyroid cancer also could be a novel biomarker for detecting thyroid malignancies.

The Enigma functions as a scaffold protein through interconnection with the cytoskeleton network and acts as an adaptor protein through the stabilization of cell membrane and signaling machinery [2,3]. The Enigma promotes heart and skeletal muscle organization during organ development and is involved in bone formation by enhancing bone morphogenetic factor-mediated osteogenesis [4]. The Enigma can increase cellular proliferation [5], suggesting it could play a role in thyroid cancer oncogenesis. The Enigma (also named PDLIM7 and LMP-4) is an intracellular non-secreted protein, composed of a PDZ and three LIM domains [2,3,4], in which the PDZ domain binds actin-binding proteins, such as β-tropomyosin, and is involved in bone development in addition to a variety of cell signaling. The LIM domain binds to tyrosine kinases [2], such as those involved in mitogenic signaling [5], including protein kinase C [6].

The direct and indirect pathways of the Enigma were shown in different cancers, including thyroid cancer. Studies have shown that the Enigma interacts with membranous Vitamin D Receptors (VDR) [7], Murine Double Minute 2 (MDM2) [8,9], phosphoinositide 3-kinase/protein kinase B (PI3K/AKT) [10,11], and bone morphogenetic protein-1 (BMP-1) [1]. The interaction of the Enigma and VDR is important as we have found that advanced thyroid cancer has low expression levels of vitamin D binding protein (DBP) in advanced thyroid cancer tissues with a stage-dependent higher expression of the Enigma protein in the corresponding thyroid cancer tissues [12]. We have shown a loss of DBP and overexpression of the Enigma in the corresponding tissues [12]. There is evidence that VDR polymorphisms may be associated with the risk and aggressive forms of thyroid cancer [13]. We also showed a differential expression of VDR (unpublished data) in thyroid cancer tissues; therefore, our lab is also focusing on VDR-Enigma interactions. We speculate that the loss of DBP enhances the vitamin D-independent function of VDR and enables the interactions of the membranous VDR to other proteins, including the Enigma.

The Enigma directly binds MDM2 as found in a study using human hepatoma and colon carcinoma cell lines demonstrated that the Enigma directly interacts with MDM2 to form a ternary complex with the p53 tumor suppressor [9]. As MDM2 is a negative regulator of p53, the upregulation of MDM2 occurs in many tumors by inactivating the apoptotic and cell cycle arrest functions of p53 [9]. The Enigma interacts with PI3K/AKT as it was shown in thyroid cancer that Enigma promoted the survival of thyroid carcinoma cells through aberrant activation of PI3K/AKT signaling [11]. Suppression of the Enigma reduces cell viability, increases the percentage of dead cells, and inactivates PI3K/AKT signaling in thyroid carcinoma cells [11]. The Enigma may also have an interaction with BMP-1 as BMP-1 is highly expressed in thyroid cancer ossification [4]. We observed a strong colocalization signal of the Enigma and BMP-1 [1] and hypothesized that the Enigma may recruit BMP-1 in malignant calcification in thyroid cancer [1].

Little is known regarding the regulation of the Enigma expression. Mapping these unknown regulatory pathways is the key to understanding the role of the Enigma in thyroid cancer. Recent studies have identified the link between microRNA (miRNA) expression and cancer, where significant changes in miRNA expression have been detected in malignant cells compared to benign cells [14]. miRNAs are involved in regulation of gene expression by targeting messenger RNA (mRNA) at the post-transcriptional level [15], and, in this way, miRNAs regulate mRNAs in cancer signaling pathways [16,17]. Improperly functioning miRNAs can alter the expression of tumor suppressor genes or oncogenes and are associated with various cancers. In normal tissues, tumor suppressor microRNAs (miRNAs) play a crucial role in preventing tumor development by suppressing the expression of oncogenes. However, during the progression of cancer, the downregulation of these miRNAs is a contributing factor that promotes cancer development. The miRNAs can regulate the oncogenes and tumor suppressor genes; therefore, they are called “oncomirs”. In cancer, oncomirs are upregulated/downregulated. They promote many cancer development via upregulating oncogenes and downregulating tumor suppressor genes, including thyroid cancer [18,19,20]. Identifying specific upregulated/downregulated miRNAs and their target genes could provide a target for future therapies.

The relationship between miRNA and PDLIM7 expression is being explored. It has been shown that impairment of miRNA-directed decay of transcription factors, such as serum response factor (SRF), increases the expression of PDLIM7 in cancer. Direct targeting of PDLIM7 mRNA by let-7 has been previously predicted [17,21,22]. In our previous study, we observed differential expression of miRNAs, including the upregulation of miR-4633-5p and downregulation of the let-7 family genes, particularly let-7g, in thyroid cancer patients from two ethnic groups, European Americans and Filipino Americans [19]. Our current objective is to investigate the correlation between PDLIM7 and let-7 gene expression using a limited amount of cancer tissue samples.

In this study, we assessed the expression levels of VDR, MDM2, PI3K/AKT, DBP, and BMP-1 through Western blotting and co-immunoprecipitation assays. We also investigated whether an inverse relationship exists between PDLIM7 and let-7g using qPCR. By determining these interactions and pathways, we aim to develop a comprehensive understanding of the Enigma’s potential as a biomarker in thyroid cancer.

## 2. Materials and Methods

### 2.1. Tumor Sample Selection

We have obtained a total of 87 deidentified and discarded formalin-fixed paraffin-embedded (FFPE) and fresh frozen tissues from thyroid cancer patients from men and women. We only selected papillary thyroid cancer (PTC) tissues to keep the uniformity. Out of 87 samples, we have eighty-four PTCs and three benign tissues as control.

### 2.2. miRNA and Total RNA Isolation from FFPE

To extract miRNA and total RNA from formalin-fixed paraffin-embedded (FFPE) samples as described before [1,19]. An AllPrep DNA/RNA FFPE Kit (QIAGEN, Valencia, CA, USA) was performed after pathological evaluation of the tumor area. The tumor tissues were processed according to the instructions of the kit (QIAGEN). We purified miRNA and total RNA with high quantity and quality. The quality and quantity were verified by using Nanodrop (NanoDrop Technologies, Waltham, MA, USA). We only used RNA with OD 260/280 < 1.8.

### 2.3. RNA and Protein Isolation from Fresh Samples

For extraction of RNA and protein from frozen fresh tissue samples in papillary thyroid cancer (PTC), we used an AllPrep^®^ DNA/RNA/Protein Mini Kit (QIAGEN, Valencia, CA, USA). We excluded other histological subtypes to maintain the uniformity. We extracted the protein for later use in Western blotting, and the RNA for RT-qPCR analysis. Each extraction process uses 0.03 g of malignant fresh thyroid tissue lysed with Buffer RLT and Beta-Mercaptoethanol, ensuring the separation of DNA, RNA, and protein. The lysate is inserted into the AllPrep DNA spin column where the genomic DNA binds to the membrane, while the RNA and protein are eluted into the flow-through. Here, 100% ethanol is combined with the DNA spin column flow-through, which is then inserted into the RNeasy spin column where total RNA binds to the membrane and the protein is eluted into the flow-through. Buffer APP is combined with the RNeasy spin column flow-through to create a precipitation of protein which is turned into pellets by centrifugation. To analyze the RNA extracted from the fresh frozen thyroid tissue samples, the quality and quantity of RNA was recorded using Nanodrop (NanoDrop Technologies, Waltham, MA, USA) [19]. RNA samples were excluded from further analysis if OD 260/280 < 1.8.

### 2.4. Western Blot Analysis and Quantitative Analysis by Densitometric Assay

Protein expression of DBP (mouse, 1:1000 dilution; Proteintech, Rosemont, IL, USA), Enigma/PDLIM7 (rabbit, 1:1000 dilution; Proteintech, Rosemont, IL, USA), MDM2 (rabbit, 1:1000 dilution; Proteintech, Rosemont, IL, USA), VDR (mouse, 1:1000 dilution; Proteintech, Rosemont, IL, USA), PI3K/AKT (rabbit, 1:1000 dilution; Proteintech, Rosemont, IL, USA), BMP-1 (mouse, 1:500 dilution; Invitrogen, Carlsbad, CA, USA), and GAPDH (rabbit, 1:1000 dilution; Cell Signaling Technologies, Danvers, MA, USA) were analyzed by Western blot analysis. Protein pellets from fresh thyroid tissue with added 5% SDS were sonicated using the Q55 Sonicator (QSonica Sonicators, Newtown, CT, USA) at 30–40% amplitude. Then, the sonicated protein underwent high-speed centrifugation. Protein concentration and purity was assessed by Nanodrop (NanoDrop Technologies, Waltham, MA, USA). Protein was separated by Novex WedgeWell 10%, Tris-Glycine, 1.0 mm, mini protein gels (Invitrogen, Carlsbad, CA, USA). Protein was then electroblotted onto 0.45 μm PVDF Transfer Membranes (Thermo Scientific, Waltham, MA, USA) using the eBlot™ L1 Fast Wet Transfer System (GenScript, Piscataway, NJ, USA), or transferred onto iBlot2 NC Mini Stacks using the iBlot2 Gel Transfer Device (both from Invitrogen, Carlsbad, CA, USA). After blocking for 1 h in Intercept^®^ (TBS) Blocking Buffer (LI-COR, Lincoln, NE, USA), each diluted primary antibody was incubated at 4 °C overnight. Immunodetection was performed using IRDye^®^ 800CW Goat anti-Rabbit IgG (1:5000), IRDye^®^ 680RD Goat anti-Rabbit IgG (1:5000), and IRDye^®^ 800CW Goat anti-Mouse IgG (1:5000) secondary antibodies (all from LI-COR, Lincoln, NE, USA). Imaging was performed using the Odyssey CLx Infrared Imaging System Model 9140 (LI-COR, Lincoln, NE, USA). The size of proteins on Western blots was identified by PageRuler™ Prestained Protein Ladder (Thermo Scientific, Waltham, MA, USA). Densitometric analysis was done using software provided by LICOR image studio to quantify the expression level using GAPDH as an internal control.

### 2.5. Immunoprecipitation and Reverse Immunoprecipitation

To perform immunoprecipitation, our bait was Enigma/PDLIM7 (rabbit, 1:1000 dilution; Proteintech, Rosemont, IL, USA), and to perform reverse immunoprecipitation, our bait was VDR (rat, Invitrogen, Carlsbad, CA, USA). In either case, when performing immunoprecipitation or reverse immunoprecipitation, protein–protein interaction was assessed between our bait and any of the following not already used as our bait: VDR (rat, Invitrogen, Carlsbad, CA, USA), Enigma/PDLIM7 (rabbit, 1:1000 dilution; Proteintech, Rosemont, IL, USA), DBP (mouse, 1:1000 dilution; Thermo Scientific, Waltham, MA, USA), MDM2 (rabbit, 1:1000 dilution; Proteintech, Rosemont, IL, USA), PI3K/AKT (rabbit, 1:1000 dilution; Proteintech, Rosemont, IL, USA), p38 MAPK (rabbit, 1:625 dilution; Invitrogen, Carlsbad, CA, USA), and BMP-1 (rabbit, 1:500 dilution; Invitrogen, Carlsbad, CA, USA). We precleared the lysates before beginning immunoprecipitation or reverse immunoprecipitation. Protein pellets from fresh thyroid tissue were sonicated in 300 μL RIPA Lysis Buffer (Immunoprecipitation Kit, Abcam, Boston, MA, USA) using the Q55 Sonicator (QSonica Sonicators, Newtown, CT, USA) at 30–40% amplitude. The sonicated protein underwent overnight incubation at 4 °C and then high-speed centrifugation. The supernatant was collected, and, for immunoprecipitation, normal rabbit serum (Thermo Scientific, Waltham, MA, USA) was added, or for reverse immunoprecipitation, normal mouse serum (Invitrogen, Carlsbad, CA, USA) was added to each sample and then they incubated on ice for 1 h. Next, 50 μL of Pierce Protein A/G Magnetic Beads (Thermo Scientific, Waltham, MA, USA) were added to each sample and they rotated for 30 min at 4 °C. After another high-speed centrifugation, the supernatant was collected and 5 μL Enigma/PDLIM7 (rabbit, Proteintech, Rosemont, IL, USA) was added when performing immunoprecipitation or 5 μL VDR (rat, Invitrogen, Carlsbad, CA, USA) was added when performing reverse immunoprecipitation. Samples incubated for 4 h at 4 °C. Then, 100 μL Pierce Protein A/G Magnetic Beads (Thermo Scientific, Waltham, MA, USA) were added and samples incubated at 4 °C overnight. The next day, after high-speed centrifugation, the beads were washed three times in 1× wash buffer made from 10× Wash Buffer (Immunoprecipitation Kit, Abcam, Boston, MA, USA) and TBS 1× (KD Medical, Columbia, MD, USA). Then, a 1× loading buffer solution was added to the beads made from Loading Buffer Dye (4×) (Lincoln, NE, USA), ddH20, and 2-Mercaptoethanol (BME) (Sigma, Burlington, MA, USA). The supernatant and bead tubes were heated at 95 °C for 10 min and loaded into two Novex WedgeWell 10%, Tris-Glycine, 1.0 mm, mini protein gels (Invitrogen, Carlsbad, CA, USA). The two gels were transferred onto iBlot2 NC Mini Stacks using the iBlot2 Gel Transfer Device (both from Invitrogen, Carlsbad, CA, USA). Running two gels at the same time and under the same conditions allowed for antibodies of similar molecular weights to be probed for on separate gel membranes. After blocking for 1 h in Intercept^®^ (TBS) Blocking Buffer (LI-COR, Lincoln, NE, USA), each diluted primary antibody was incubated at 4 °C overnight. Immunodetection was performed using IRDye^®^ 800CW Goat anti-Rabbit IgG (1:5000), IRDye^®^ 680RD Goat anti-Rabbit IgG (1:5000), and IRDye^®^ 800CW Goat anti-Mouse IgG (1:5000) secondary antibodies (all from LI-COR, Lincoln, NE, USA). Imaging was performed using the Odyssey CLx Infrared Imaging System Model 9140 (LI-COR, Lincoln, NE, USA). The size of proteins on Western blots was identified by PageRuler™ Prestained Protein Ladder (Thermo Scientific, Waltham, MA, USA).

### 2.6. RT-qPCR (Real-Time Polymerase Chain Reaction)

In preparation for RT-qPCR, adapters were ligated sequentially to the 3′ and 5′ ends of RNA, then cDNA was synthesized [18]. Primers used include PDLIM7 (forward 5′- CAG AGC CGC ACC TCC ATT G -3′ and reverse 5′- TGG TGA CAC ACG GGA GTC T -3′, Integrated DNA Technologies, Coralville, IA, USA) and GAPDH (forward 5′-GTC TCC TCT GAC TTC AAC AGC G-3′ and reverse 5′-ACC ACC CTG TTG CTG TAG CCA A-3′, Integrated DNA Technologies, Coralville, IA, USA). Then, RT-qPCR allowed for amplification using the following conditions: 95 °C 15 min; 13 cycles of (95 °C 15 s, 60 °C 30 s, and 72 °C 15 s); 72 °C 2 min [19]. The ∆∆ Ct method, also known as the 2^–∆∆Ct^ method, is a simple formula used in order to calculate qPCR. Ct stands for the cycle threshold (Ct) of the sample. This is given after the qPCR reaction by the machine. It is the cycle number where the fluorescence is generated by the PCR. The symbol **∆** refers to delta. Delta is a mathematical term used to describe the difference between two numbers. So, it is useful to use when summarizing long formulas. U6 was used for miR normalization. The results were analyzed using the ΔΔ cycles to threshold (ΔΔCt).

**∆∆Ct = ∆Ct (gene of interest) − ∆Ct (control).**

### 2.7. Statistical Analysis

All statistical analysis was performed using SAS software 9.4 version and included the whole sample of 84 observations. The statistical methods were employed to understand association and provide valuable insights into the strength and direction of relationships, aiding in the comprehensive understanding of inter-variable associations within our dataset. To determine the relationships between the variables ∆∆ct_PDLIM7 and tumor staging, Pearson and Spearman rank correlation coefficients were applied. Univariate analysis was carried out to analyze each of the gene expressions. This analysis provided us with means, standard deviation (SD), student’s t statistics, quartiles, and histograms for each of the gene expressions. Following this *t*-test was performed to confirm difference in means and standard deviations with 95% confidence intervals between PDLIM7 and let-7 gene expression. The *Pearson correlation coefficient* was used to measure the strength of a linear association between PDLIM7 and let-7 gene expressions.

## 3. Results

### 3.1. Differential Expressions of Enigma and Its Signaling Pathways in Thyroid Cancer Tissues

**Figure 1:**
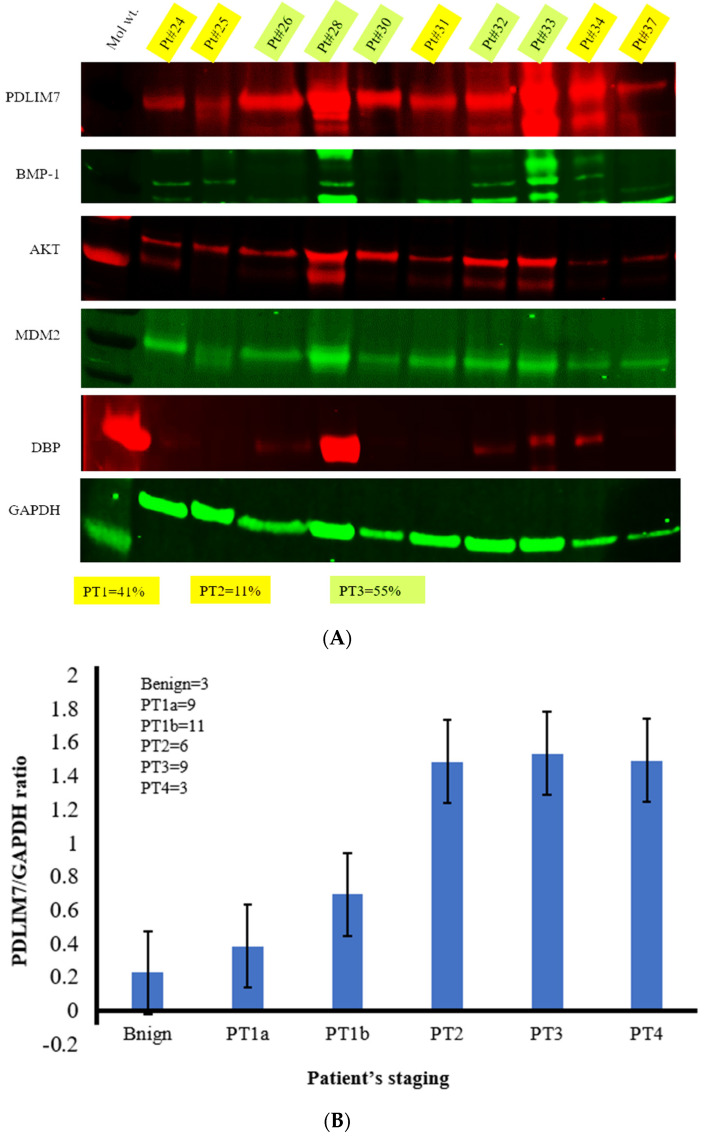
(**A**) Western blotting data of representative protein tissue samples from patients probed with Enigma (55 kDa), BMP-1 (80–100 kDa), PI3K/AKT (56 kDa), MDM2 (75–85 kDa), DBP (52–58 kDa), and GAPDH (37 kDa) antibodies in 41 cancer tissue samples. Pt#, patient number; Mol wt, molecular weight. (**B**) Densitometric analysis of PDLIM7 protein expression in different staging. B9, benign (*n* = 3), PT1a (*n* = 9); PT1b (*n* = 11); PT2 (*n* = 6); PT3 (*n* = 9); PT4 (*n* = 3). Data was shown as Mean +/− Stdev. pT1aN0-pT1bN0 (Stage I), pT1b pN1a (Stage II), pT1a(m) N1a Mx (Stage III), pT3N1bM1~ (stage IV) [1,23]. Stage I (*n* = 20); Stage II (*n* = 6); Stage III (*n* = 9); Stage IV (*n* = 3).

We observed distinct protein bands and conducted densitometric analysis to confirm the presence of proteins and quantify their relative expression levels (Figure 1A). PTNM classification as provided: P (pathological); T (tumor): T1, ≤10 mm; T2, 10–40 mm; T3, >40 mm; T4, extrathyroidal. N (node): N0, node negative; N1, node positive. M (metastasis): M0, no distant metastasis; M1, distant metastasis. pT1aN0-pT1bN0 (Stage I), pT1b pN1a (Stage II), pT1a(m) N1a Mx (Stage III), pT3N1bM1~ (stage IV).

In this study, we analyzed 41 patient samples, utilizing two separate membranes to accommodate four primary antibodies on each membrane. The results demonstrated the expression levels of Enigma, DBP, BMP-1, MDM2, PI3K/AKT, and VDR, with GAPDH serving as a control reference (Figure 1A and Supplementary Figure S1A). Initially, the study included 50 samples. However, four patients were excluded due to insufficient protein concentrations, preventing their processing on a gel. Additionally, two patients were excluded because GAPDH was absent in their samples post-gel electrophoresis, despite having adequate protein concentrations. Consequently, the total number of patients for analysis was reduced to 41. Densitometric analysis of the Enigma revealed quantitative values among the 44 patients and ratio was determined GAPDH value as control. Results showed as follows: 2-fold in PT1a (*n* = 9), 3-fold in PT1b (*n* = 11), 8-fold in PT2 (*n* = 6) and PT3 (*n* = 9), and finally 7-fold in PT4 (*n* = 3) above the control (benign) (Figure 1B). PT1a and PT1b showed no difference; however, although PT2 showed a 2–1.5 fold higher than PT1-1b, no difference was observed among PT2-PT4. The density analysis of PI3K/AKT, BMP-1, DBP, VDR, and MDM2 are shown in Supplementary Figure S1B–F.

### 3.2. Interacting Partners of Enigma in Thyroid Cancer

**Figure 2:**
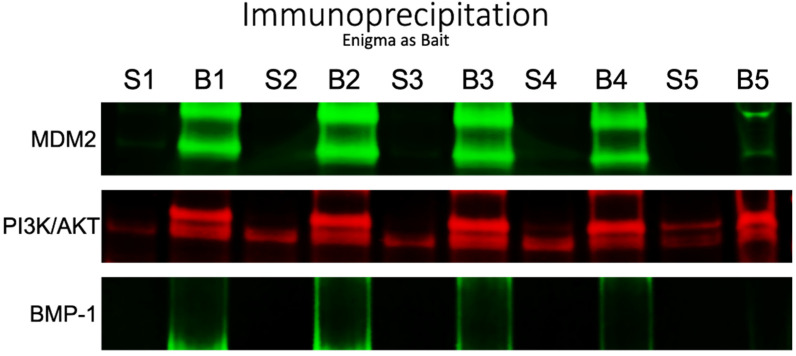
Immunoprecipitation scans of patients #1–5 showing interaction levels of MDM2, PI3K/AKT, and BMP-1 with the Enigma antibody as a bait. Two lanes per patient are shown, S (supernatant) and B (beads).

To assess potential interacting partners, we performed immunoprecipitation with the Enigma antibody (monoclonal PDLIM7 antibody) as a bait and Western blotting with several other known oncogenic proteins. Among the various known interacting partners examined, our results indicated that the Enigma interacted with MDM2 and PI3K/AKT, but not BMP-1 (Figure 2).

**Figure 3:**
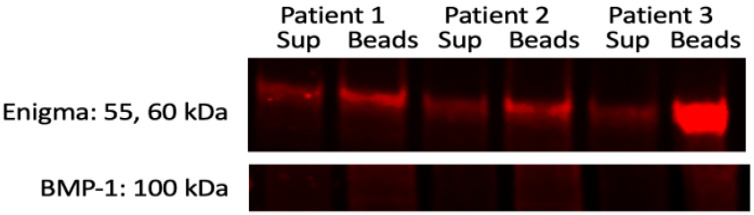
Reverse Immunoprecipitation with VDR antibody, probed with the Enigma, and BMP-1 antibodies.

Reverse immunoprecipitation study showed a strong interaction of VDR with the Enigma protein but not with BMP-1 (Figure 3). VDR also interacted with DBP.

### 3.3. Quantitative Analysis of PDLIM7 and Let-7g Gene in the Same Thyroid Cancer Patient Samples

**Figure 4:**
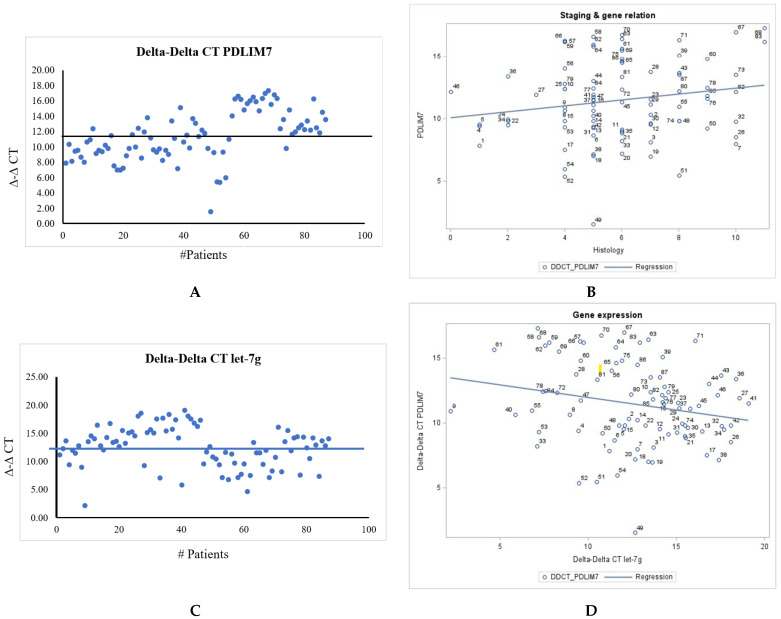
(**A**) D-D CT value of PDLIM7 expression (*Y*-axis) gene was plotted in 87 samples. (**B**) Scatter plot of D-D CT value of PDLIM7 (*n* = 84) to staging. There is a positive correlation. PT1 = 37; PT2 = 15; PT3 = 22; PT4 = 9). PTNM classification was shown in result section [1,23]. (**C**) ∆-∆ CT values of let-7g expression (*Y*-axis) gene were plotted in 87 samples. (**D**) Scatter plot of correlation between both gene expressions.

We were also able to perform RT-qPCR on a total of 87 tissue samples with the primers U6 Control, PDLIM7, and let-7g genes in triplicates. D-D CT values showed PDLIM7 expression levels (*n* = 87) compared with U6 control as shown in Figure 4A. Scatter plot of D-D CT value of PDLIM7 (*n* = 84) to staging was shown in Figure 4B. There is a positive correlation. PT1 = 37; PT2 = 15; PT3 = 22; PT4 = 9).

TCGA data analysis using UALCAN (http://ualcan.path.uab.edu/analysis.html, accessed on 27 November 2023) analysis showed a significant enhanced expression of PDLIM7 gene when compared to control (Supplementary Figure S2A). A stage-dependent enhanced expression of PDLIM7 gene was observed (Supplementary Figure S2B) with higher expression correlated with poor survival compared to low expression with better survival rate (Supplementary Figure S2C).

The Pearson correlation coefficient (r = 0.179) revealed a weak positive linear relationship, indicating a tendency for the variables to increase together, although the strength of this linear association was modest. Concurrently, the Spearman rank correlation coefficient (ρ = 0.145) showed a weak positive monotonic relationship, emphasizing that the variables tend to move together in a consistent direction without requiring a strictly linear pattern (Table 1). These findings suggest a subtle and non-linear association between the D-DCT_PDLIM7 and staging variables, underscoring the importance of considering both linear and non-linear aspects when exploring their relationship.

We were also able to perform RT-qPCR on a total of eighty-four tissue samples and three benign tissues with the primers U6 Control and let-7g in triplicates. The D-D CT values showed let-7g expression levels compared to the U6 control (Figure 4C).

Univariate analysis for let-7 gene expression produced means of 12.6 and SD ±3.6 with student’s *t*-test of 32.9 whereas for PDLIM7 gene expression means were 11.5 and SD ±3.2 with student’s *t*-test statistics as 33.7, thus providing significant insight into their statistical characteristics. Individual *t*-tests from univariate analysis on let-7 gene and PDLIM7 gene were done to assess whether each group’s mean was different and to examine their distribution individually. Histograms for both gene expressions were right skewed as seen in Supplementary Figure S3A–C and these visualizations revealed patterns, that the gene expressions data follow a right skewness or kurtosis characteristics. Identifying and handling outliers within each gene expression variable is essential, therefore univariate analysis helped identify those outliers. The *t*-test value of 1.96 suggests that this difference is statistically significant, and with 95%CI between (4.58, 6.18) suggesting that the true population parameter falls within the range, further suggesting a statistically significant difference in both gene expressions. Therefore, conducting a univariate analysis and *t*-test on these gene expression values for 87 subjects was done to investigate for both gene expressions having possibility of being associated with thyroid cancer progression. Pearson’s correlation coefficients for 87 tissue samples was −0.217, which suggests that there is a very weak, negative linear association between the two gene expressions but still follows a linear pattern (Figure 4D).

The student’s *t*-test results are noteworthy as they indicate a mean difference of 1.10 between D-D let-7g and PDLM7 (Table 2). It also indicates that while one gene expression increases the other decreases and vice versa. However, further analysis can be done to understand the nature of the relationship and any potential underlying factors.

Pearson’s correlation analysis was operated to uncover the correlation pair between differentially expressed cancer-associated PDLIM7 and let-7g gene, whose correlation coefficient was −0.217, and *p* < 0.05 (Figure 4D).

## 4. Discussion

Our comprehensive evaluation of the Enigma, including its associated pathways and interactions, adds depth to the understanding of this protein’s role in thyroid cancer. The exploration of the Enigma’s associated pathways and interactions with proteins such as VDR, MDM2, PI3K/AKT, and BMP-1 is consistent with previous studies. The hypothesis linking reduced DBP levels to increased Enigma expression is intriguing. The DBP density analysis, showing varying expression levels, provides further insight into potential mechanisms underlying the Enigma expression regulation. We observed a trend of a stage-dependent higher expression of PDLIM7 gene by qPCR assay from a small tissue sample. The limiting factor of our study was a lack of tissue samples in different staging of PTCs. We also see a significant but not a strong correlation between PDLIM7 and miR-let-7g gene in thyroid cancer tissues.

To gain an understanding of the Enigma as a potential thyroid cancer biomarker, we conducted a thorough evaluation of its associated pathways and interactions. The existing literature highlights the Enigma’s interactions with various proteins, including VDR, MDM2, PI3K/AKT, and BMP-1. Within our study, quantitative analysis of the Enigma’s expression distribution aligned with our previous immunohistochemical analysis [1], we have also shown an association between low DBP levels and increased Enigma expression [13].

The assessment of VDR density and its distribution of expression levels, from low to high, adds another layer to the proposed mechanism involving the Enigma and VDR.

Elevated MDM2 expression is a recognized phenomenon in various tumors, contributing to the suppression of p53’s apoptotic and cell cycle arrest functions. A study demonstrated a direct interaction between the Enigma and MDM2, forming a complex involving p53. Interestingly, the Enigma co-expresses with MDM2 but not p53 in certain liver and stomach tumors [9]. Our prediction aligned with an increase in MDM2 expression. The density analysis of MDM2 revealed a distribution of expression levels from low to high.

The Enigma’s role in promoting the survival of thyroid carcinoma cells by activating the PI3K/AKT signaling pathway has been documented [11]. In the context of cancer, the PI3K/AKT pathway is crucial for cell survival under stress conditions [10]. Our initial expectations aligned with an increase in PI3K/AKT expression. However, density analysis of PI3K/AKT revealed a differential expression level, indicates variability in its activation across thyroid cancer samples.

BMP-1, belonging to the BMP family, is known for its role in converting precursor proteins into active forms, participating in functions like cell adhesion and the regulation of mineralization [1]. BMP-1 expression has been observed in thyroid cancer-related ossification [4]. Our hypothesis was that the Enigma could interact with BMP-1 in the context of malignant calcification in thyroid cancer [1]. Previous research identified a strong colocalization signal between the Enigma and BMP-1 [1]. The colocalization without a demonstrated interaction was also found in our study. Our findings suggest expression of the Enigma and BMP-1 may promote different pathways within thyroid cancer tissue. These unexpected findings open avenues for further research to elucidate the factors influencing the expression of these proteins and their interplay in thyroid carcinoma.

The observation of variable expression levels of the PDLIM7 gene in thyroid cancer tissues suggests heterogeneity within the cancer samples. Understanding this variability is crucial for deciphering the role of PDLIM7 in thyroid cancer progression. Our current finding of the Enigma’s quantitative expression distribution is consistent with our previous immunohistochemical analysis and TCGA data reinforces the reliability of our findings and adds consistency to our results.

Differential expressions of miRNAs were reported in thyroid cancer [18,19,20,21,22]. microRNAs, particularly the let-7 family, and their potential role in thyroid cancer development reveals the complexity of molecular regulation in cancer. These miRNAs play a crucial role in inhibiting the translation of several oncogenes, such as Myc, K-Ras, and HMGA2. We observed a very weak, negative linear association between let-7g and PDLIM7 gene expressions, as suggested by Pearson’s correlation. However, the statistical significance indicates a potential relationship between the dysregulation of let-7g and the expression of PDLIM7 in thyroid cancer. Exploration of molecular and cellular mechanisms may discover the additional interaction between let-7g and PDLIM7 in thyroid cancer.

We have switched a diagnostic approach using a quantitative qPCR assay instead of an immunohistochemical study of the Enigma protein expression to detect the differential expression of the PDLIM7 gene. qPCR has advantages, particularly in terms of time efficiency and potential applicability in presurgical evaluation of thyroid nodules. qPCR is known for its quantitative accuracy and sensitivity in measuring gene expression levels. Understanding PDLIM7 gene expression levels could provide insights into the differentiation between early and advanced stages of thyroid cancer. The use of qPCR has advantages for its time efficiency compared to immunohistochemistry. This can be particularly valuable in clinical settings where quick and reliable diagnostic information is essential for decision-making. Rapid qPCR of a small tissue specimen from a fine needle aspiration could have significant clinical utility.

In conclusion, a rapid PDLIM7-qPCR of a small tissue specimen from a fine needle aspiration would have significant clinical utility. Our research has provided additional valuable insights into the potential correlation between PDLIM7 and let-7g genes in thyroid cancer, as well as a detailed exploration of the Enigma and its associated pathways. The integration of various analytical techniques enhances the depth and reliability of our study, laying the groundwork for further investigations and potential clinical implications in thyroid cancer diagnosis and treatment.

## Figures and Tables

**Table 1:** Output from Pearson and Spearman correlation and its explanation between variables ∆-∆ CT_PDLIM7 and tumor staging.

Pearson Correlation Coefficients, N = 87 Prob > |r| under HO: Rho = 0		
	**DDCT_PDLIM7**	**Staging**
DDCT_PDLIMZ	1.00000	0.17866
DDCT_PDLIM7		0.0978
Staging	0.17866	1.00000
Staging	0.0978	
**Spearman Correlation Coefficients, N = 87** **Prob > |r| under HO: Rho = 0**		
	**DDCT_PDLIM7**	**Staging**
DDCT_PDLIM7	1.00000	0.14464
DDCT_PDLIM7		0.1813
Staging	0.14464	1.00000
Staging	0.1813	

**Table 2:** *t*-test Results showing difference in means between DDCT_let-7g and DDCT_PDLIM7.

N	*t*-Value	Mean & SD	95% CI
87	1.96	1.10 ± 5.26	(4.58, 6.18)

## Data Availability

The data presented in this study is available in this article (and Supplementary Material).

## Supplementary Materials

The following supporting information can be downloaded at: https://www.mdpi.com/article/10.3390/curroncol30120761/s1, Figure S1A. Western blotting of representative samples of 9 patients probed with Enigma (55 kDa); Figure S1B. Densitometric analysis of Western blot images using LICOR Image Studio 5.5. The DBP/GAPDH ratio was calculated from the raw data in 44 patients; Figure S1C. Densitometric analysis of Western blot images using LICOR image software. The VDR/GAPDH ratio was calculated from the raw data in 44 patients: Figure S1D Densitometric analysis of Western blot images using LICOR image software. The BMP-1/GAPDH ratio was calculated from the raw data in 44 patients; Figure S1E. Densitometric analysis of Western blot images using LICOR image software. The PI3K/AKT/GAPDH ratio was calculated from the raw data in 44 patients; Figure S1F. Densitometric analysis of Western blot images using LICOR image software. The MDM2/GAPDH ratio was calculated from the raw data in 44 patients. Figure S2A. TCGA analysis of PDLIM7 gene expression in normal (*n* = 59) versus primary tumor (*n* = 505); Figure S2B. TCGA analysis of PDLIM7 gene expression in normal (*n* = 59) versus Stage 1 (*n* = 284), Stage 2 (*n* = 52), Stage 3 (*n* = 112), and Stage 4 (*n* = 55); Figure S2C. TCGA analysis of PDLIM7 gene expression on patient’s survival (high expression, *n* = 127), (low expression, *n* = 377). Figure S3A. Histogram of PDLIM-7 gene expression; Figure S3B. Histogram of let-7 gene expression; Figure S3C. Histogram showing the difference between both gene expressions.
