# Supplementary material for: Regulatory and Interacting Partners of PDLIM7 in Thyroid Cancer
Source: Curr Oncol. 2023 Dec 13;30(12):10450–62. doi: 10.3390/curroncol30120761 (PMC10742985; doi:10.3390/curroncol30120761)
Supplement: Supplementary file 1 [file curroncol-30-00761-s001.zip › curroncol-2621579-supplementary.pdf]

Supplemental Figures:

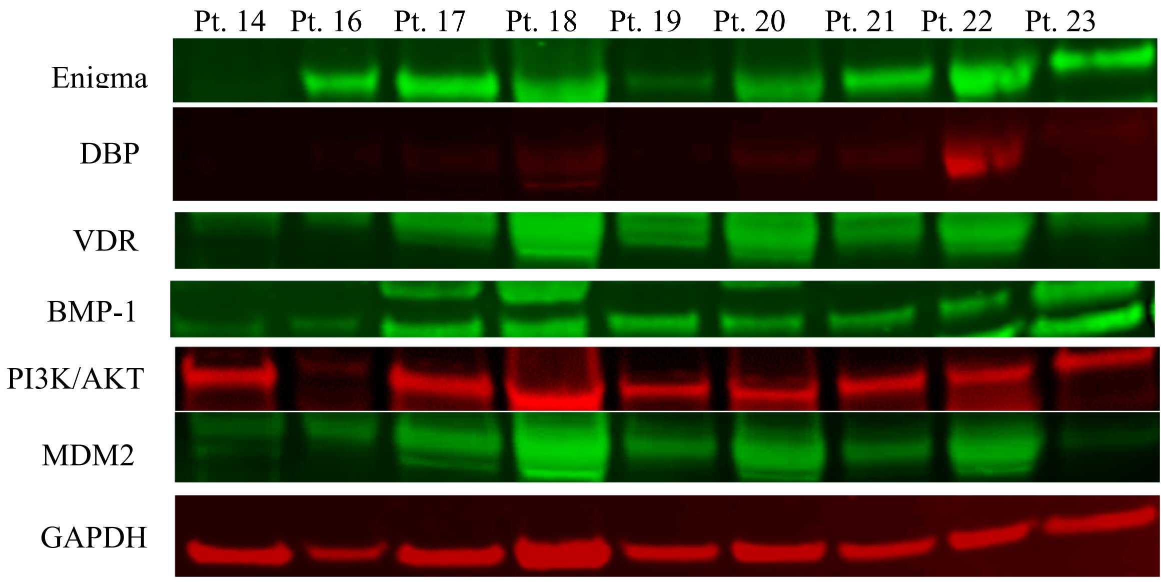

Supplemental Figure S1A*. Western blotting of representative samples of 9 patients probed with Enigma (55 kDa), BMP-1 (80-100 kDa), DBP (52-58 kDa), VDR (48-55), PI3K/AKT (56 kDa), MDM2 (75-85 kDa), and GAPDH (37 kDa) antibodies (n=44 tissue samples). Pt, patient.*

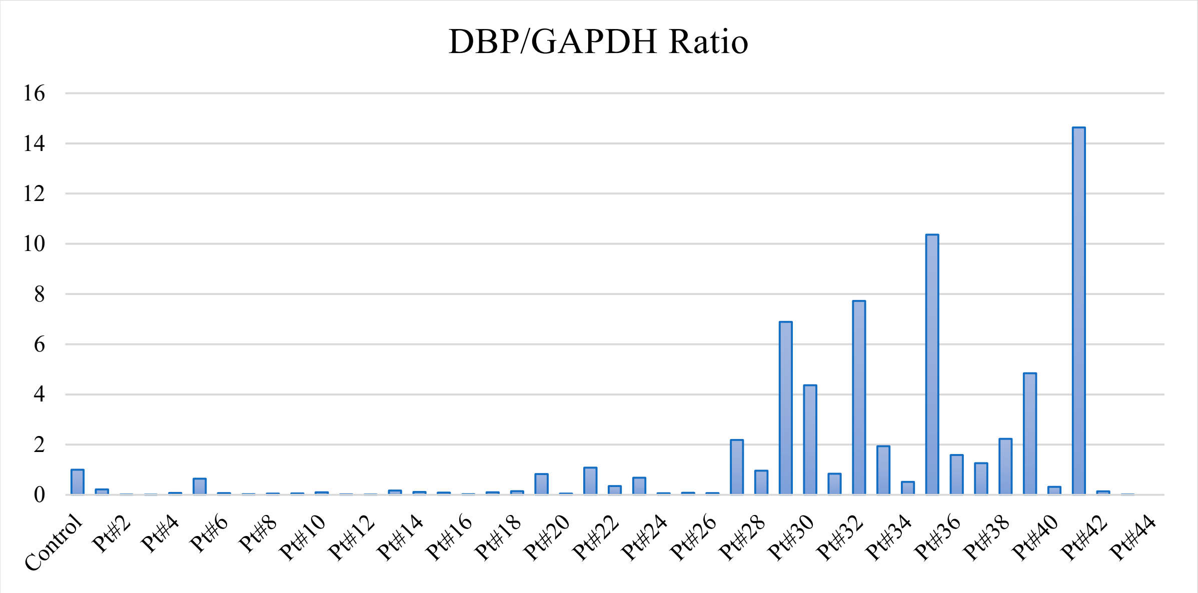

Supplemental Figure S1B*. Densitometric analysis of western blot images using LICOR image software. The DBP/GAPDH ratio was calculated from the raw data in 44 patients.*

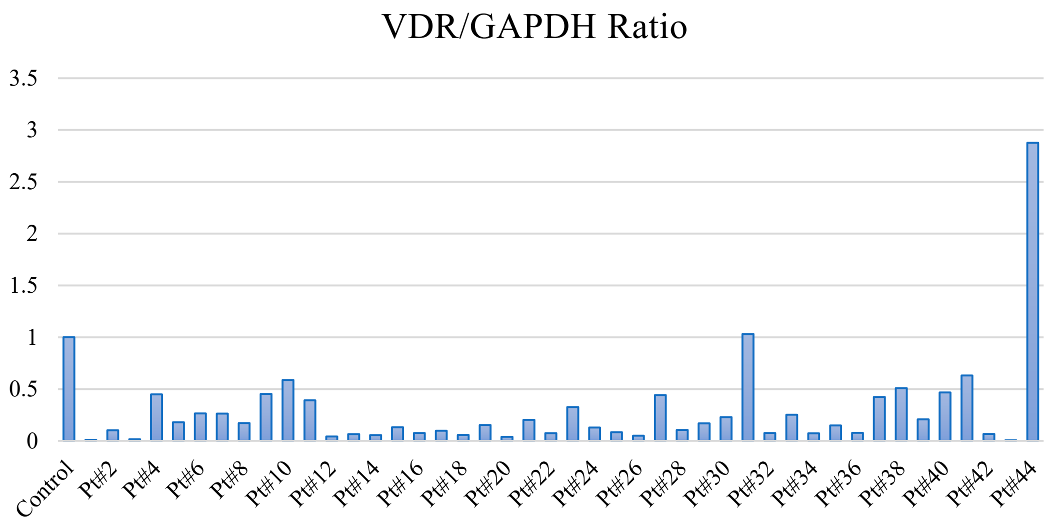

Supplemental Figure S1C*. Densitometric analysis of western blot images using LICOR image software. The VDR/GAPDH ratio was calculated from the raw data in 44 patients.*

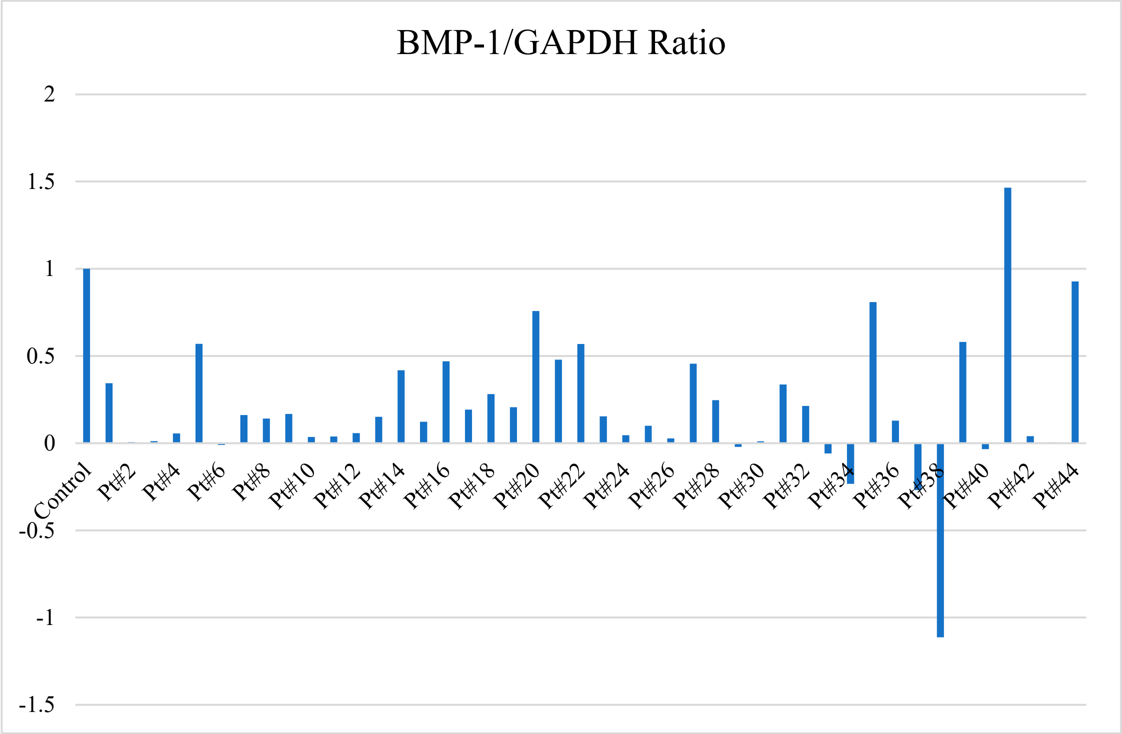

Supplemental Figure S1D*. Densitometric analysis of western blot images using LICOR image software. The BMP-1/GAPDH ratio was calculated from the raw data in 44 patients.*

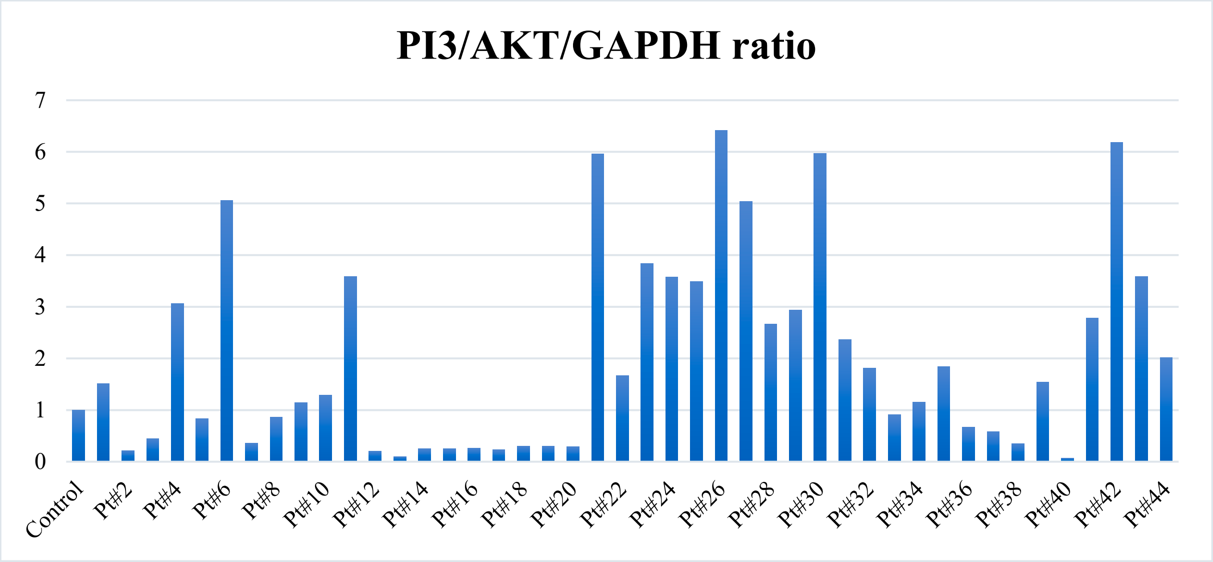

Supplemental Figure S1E*. Densitometric analysis of western blot images using LICOR image software. The PI3K/AKT/GAPDH ratio was calculated from the raw data in 44 patients.*

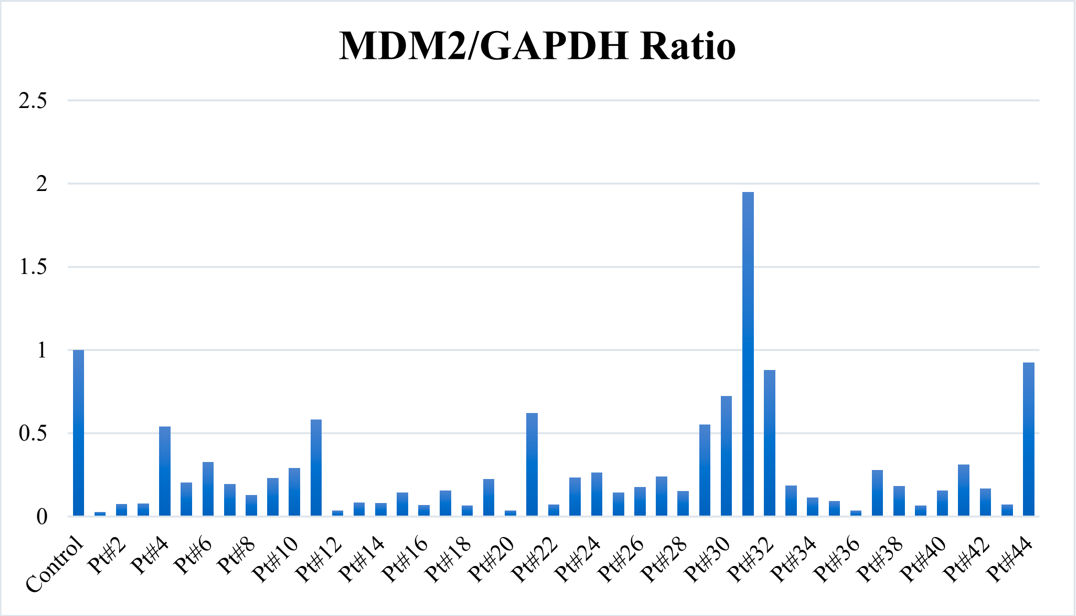

Supplemental Figure S1F*. Densitometric analysis of western blot images using LICOR image software. The MDM2/GAPDH ratio was calculated from the raw data in 44 patients.*

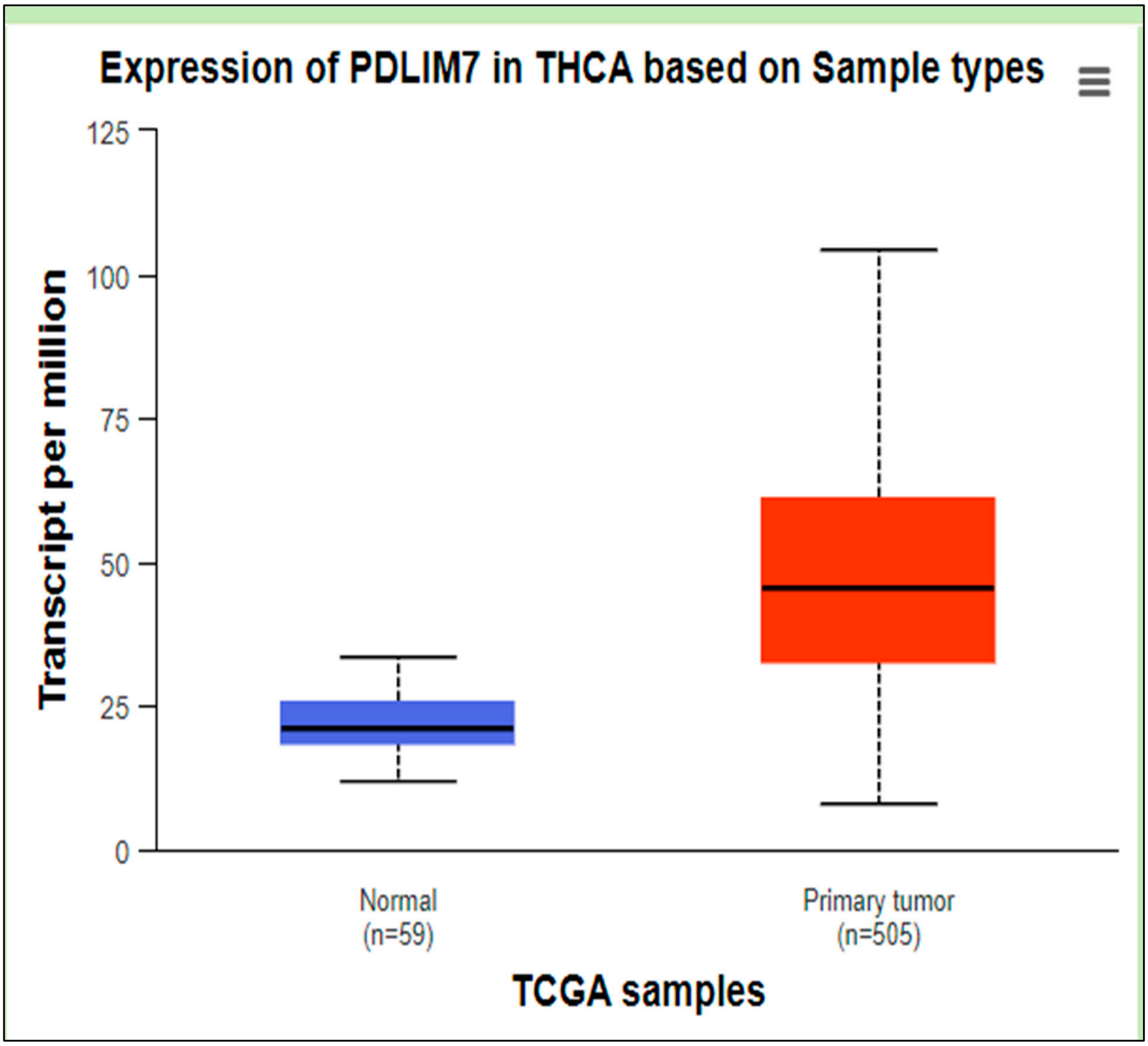

Supplemental Figure S2A. *TCGA analysis of PDLIM7 gene expression in normal (n=59) versus primary tumor (n=505).*

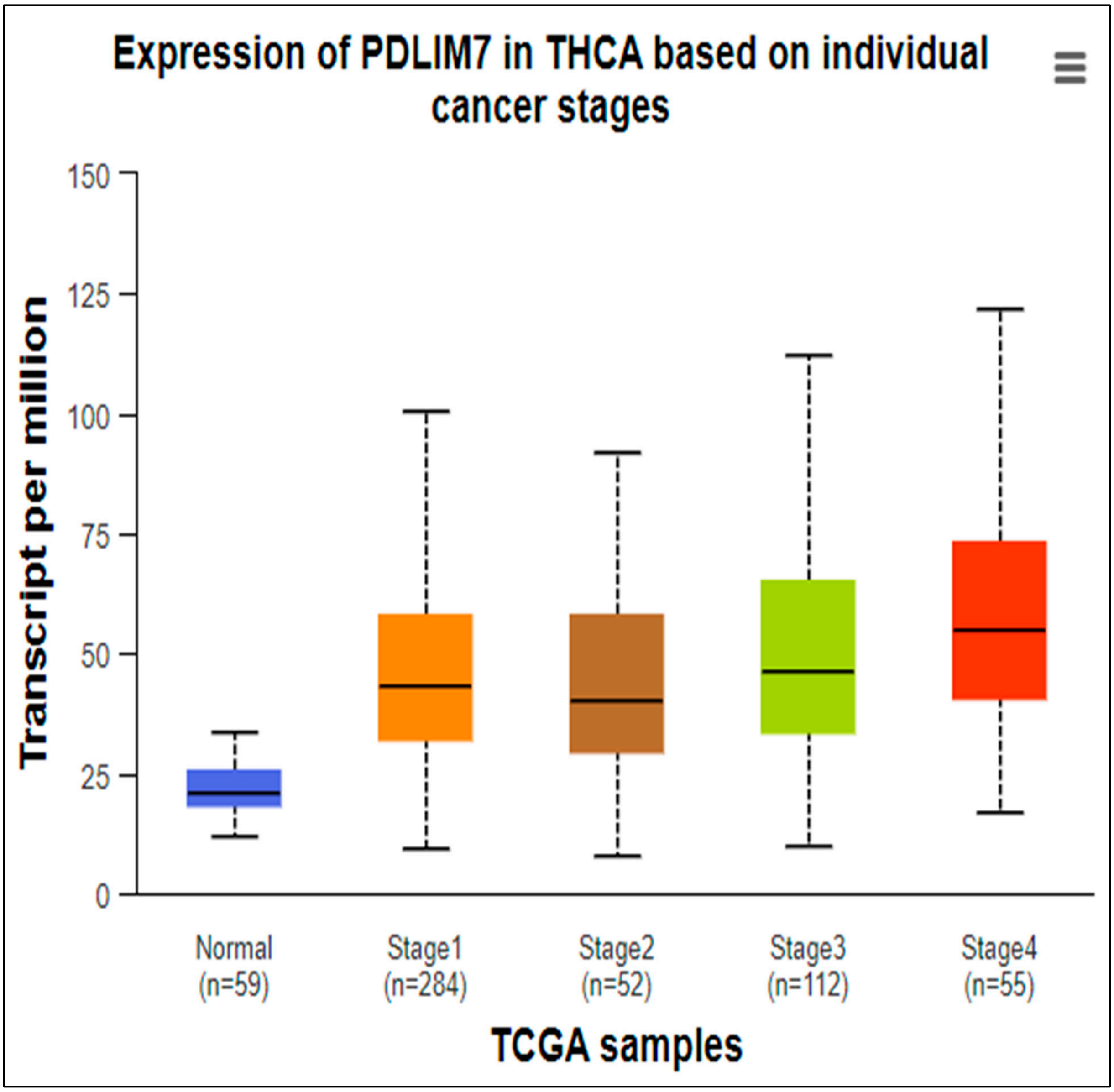

Supplemental Figure S2B. *TCGA analysis of PDLIM7 gene expression in normal (n=59) versus Stage 1(n=284), Stage 2(n=52), Stage 3(n=112), and Stage 4 (n=55).*

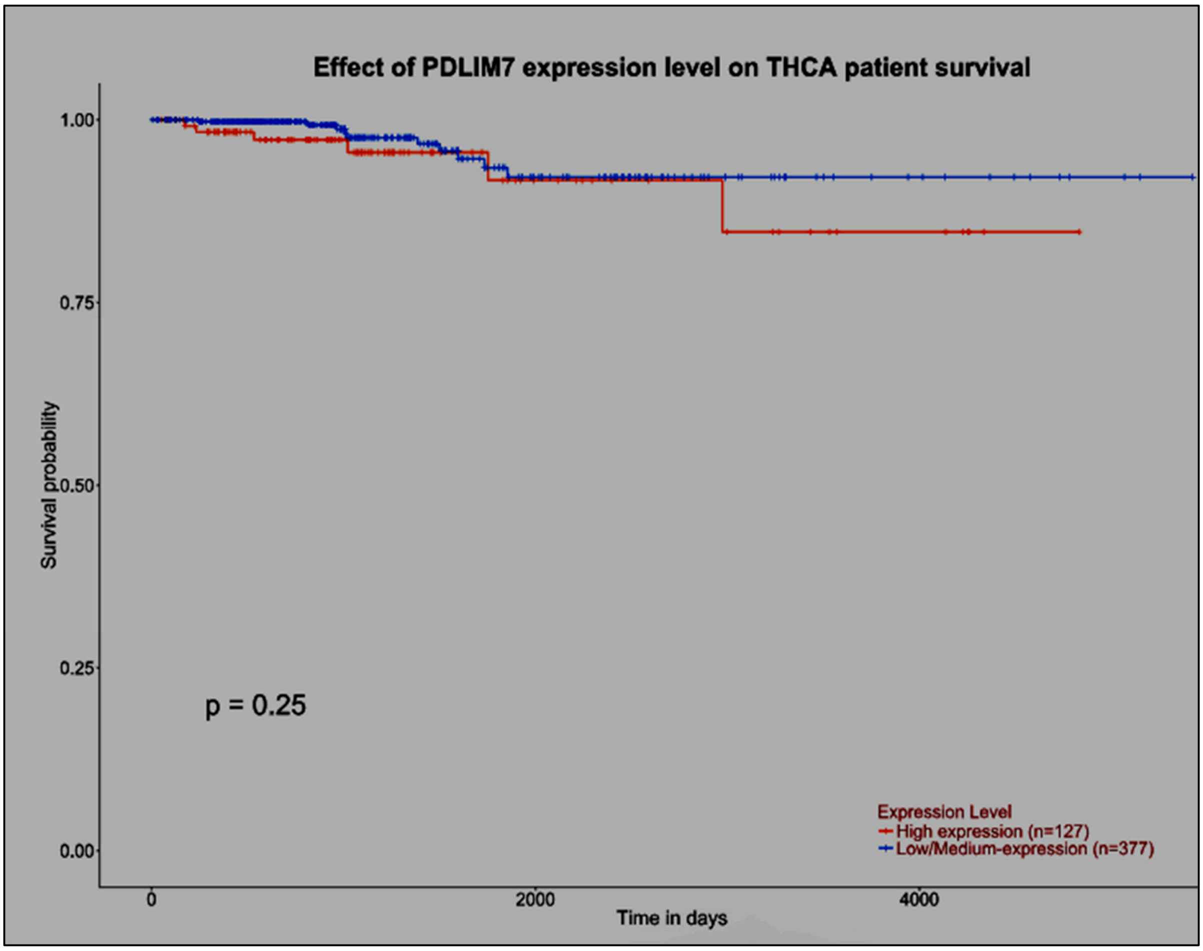

Supplemental Figure S2C*. TCGA analysis of PDLIM7 gene expression on patient's survival (high expression, n=127), (low expression, n=377).*

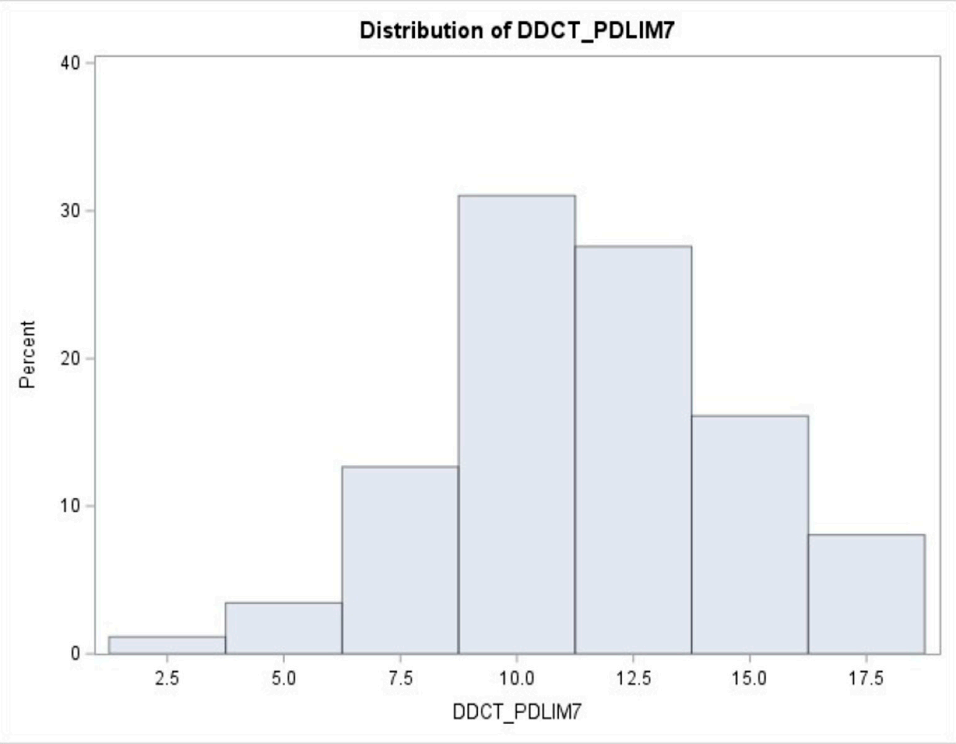

Supplemental Figure S3A. *Histogram of PDLIM-7 gene expression*

Gene expression

The UNIVARIATE Procedure

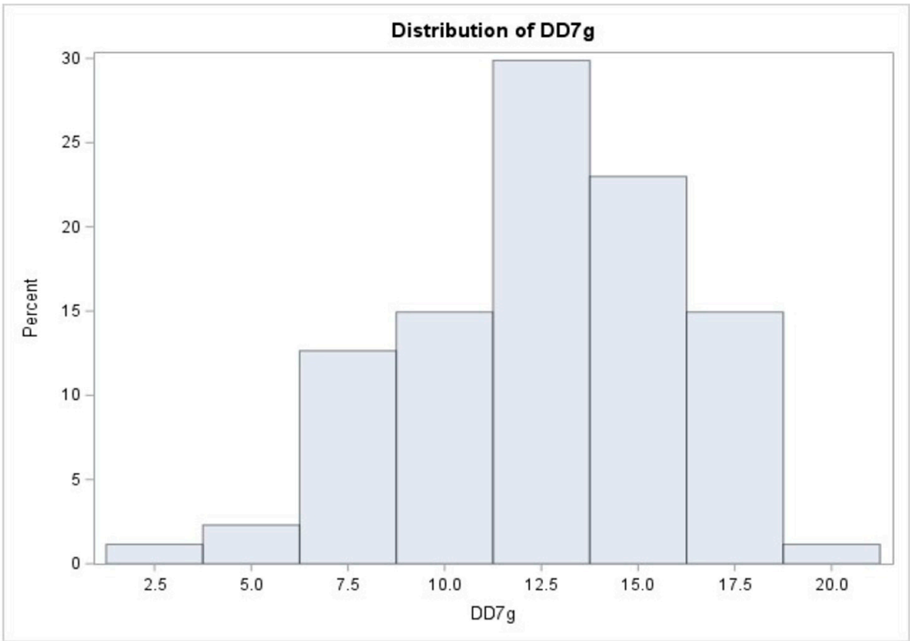

Supplemental Figure S3B. *Histogram of let-7 gene expression.*

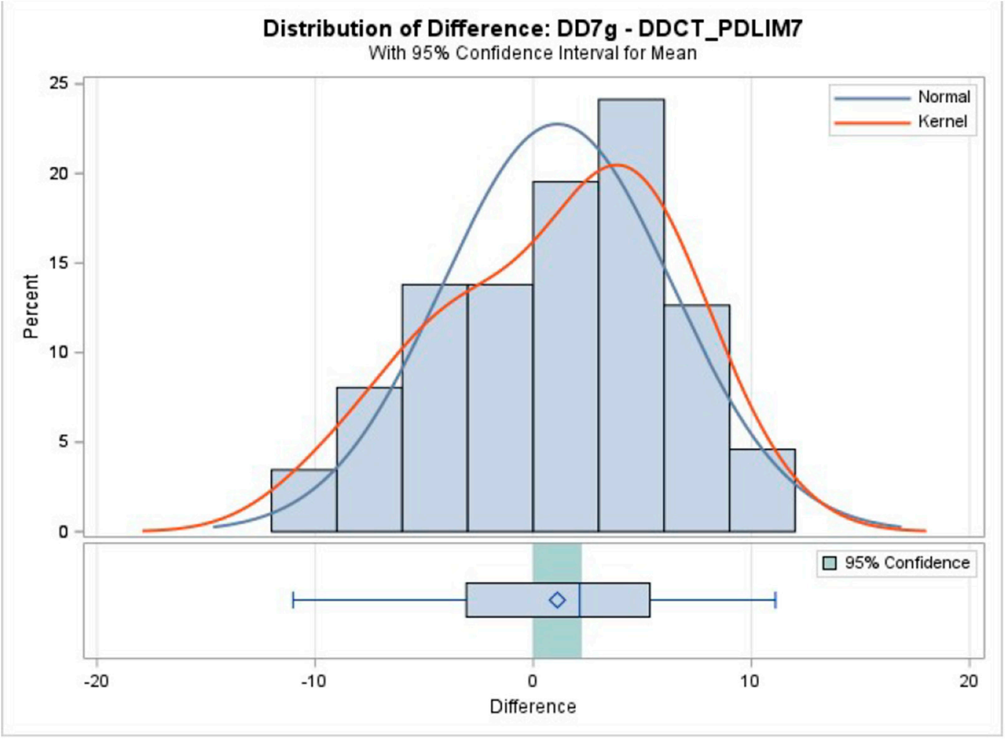

Supplemental Figure S3C. *Histogram showing the difference between both gene expressions.*
